# Effect of Dexmedetomidine on Postpartum Depression in Women With Prenatal Depression: A Randomized Clinical Trial

**DOI:** 10.1001/jamanetworkopen.2023.53252

**Published:** 2024-01-25

**Authors:** Yingyong Zhou, Zhihong Bai, Wenchao Zhang, Shouyu Xu, Yunfei Feng, Qiuwen Li, Lishan Li, Anqi Ping, Liang Chen, Saiying Wang, Kaiming Duan

**Affiliations:** 1 Department of Anesthesiology, The Third Xiangya Hospital of Central South University, Changsha, China; 2 Department of Anesthesiology, Beijing Jishuitan Hospital, Capital Medical University, Beijing, China; 3 Department of Anesthesiology, The Maternal and Child Health Hospital of the Hu Nan Province, Changsha, China

## Abstract

**Importance:**

Postpartum depression (PPD) is emerging as a major public health problem worldwide. Although the particular period and context in which PPD occurs provides an opportunity for preventive interventions, there is still a lack of pharmacologic prevention strategies for PPD.

**Objective:**

To assess the efficacy and safety of dexmedetomidine for prevention of PPD among women with prenatal depression undergoing cesarean delivery.

**Design, Setting, and Participants:**

This randomized clinical trial enrolled 338 women who screened positive for prenatal depression at 2 hospitals in Hunan, China from March 28, 2022, to April 16, 2023. Women with an Edinburgh Postnatal Depression Scale score of more than 9 who were 18 years of age or older and were scheduled for elective cesarean delivery were eligible.

**Interventions:**

Eligible participants were randomly assigned in a 1:1 ratio to either the dexmedetomidine group or the control group via centrally computer-generated group randomization. Dexmedetomidine, 0.5 μg/kg and 0.9% saline were intravenously infused for 10 minutes after delivery in the dexmedetomidine and control groups, respectively. After infusion, sufentanil or dexmedetomidine plus sufentanil was administered via patient-controlled intravenous analgesia for 48 hours in the control group and dexmedetomidine group, respectively.

**Main Outcomes and Measures:**

The primary outcome was positive PPD screening results at 7 and 42 days post partum, defined as a postpartum Edinburgh Postnatal Depression Scale score of more than 9. Analysis was on an intention-to-treat basis.

**Results:**

All 338 participants were female, with a mean (SD) age of 31.5 (4.1) years. Positive PPD screening incidence at 7 and 42 days post partum in the dexmedetomidine group vs the control group was significantly decreased (day 7, 21 of 167 [12.6%] vs 53 of 165 [32.1%]; risk ratio, 0.39 [95% CI, 0.25-0.62]; *P* < .001; day 42, 19 of 167 [11.4%] vs 50 of 165 [30.3%]; risk ratio, 0.38 [95% CI, 0.23-0.61]; *P* < .001). The dexmedetomidine group showed no significant difference in adverse events vs the control group (46 of 169 [27.2%] vs 33 of 169 [19.5%]; *P* = .10), but the incidence of hypotension increased (31 of 169 [18.3%] vs 16 of 169 [9.5%]; risk ratio, 2.15 [95% CI, 1.13-4.10]; *P* = .02).

**Conclusions and Relevance:**

Dexmedetomidine administration in the early postpartum period significantly reduced the incidence of a positive PPD screening and maintained a favorable safety profile.

**Trial Registration:**

Chinese Clinical Trial Registry Identifier: ChiCTR2200057213

## Key Points

**Question:**

Does dexmedetomidine administration in the early postpartum period prevent postpartum depression (PPD) among women with prenatal depression?

**Findings:**

In this randomized clinical trial of 338 women with prenatal depression, preventive administration of dexmedetomidine in the early postpartum period significantly reduced the incidence of PPD and postoperative pain, while also improving sleep quality.

**Meaning:**

This study found that preventive administration of dexmedetomidine in the early postpartum period reduced the incidence of PPD.

## Teaser

This randomized clinical trial assesses the efficacy and safety of dexmedetomidine for prevention of postpartum depression among women with prenatal depression undergoing cesarean delivery.

## Introduction

Postpartum depression (PPD) is the most common psychological disorder among women after birth, with an incidence of about 10% to 20%.^1,2,3^ Given the serious negative consequences for mothers, young children, family members, and society, strategies to improve PPD prevention and treatment are a public health priority.^4^ The US Preventive Services Task Force report provides evidence indicating that depression screening and intervention for pregnant and postpartum women can reduce depressive symptoms and increase the likelihood of remission.^5^ Prenatal depression is a PPD risk factor,^6,7^ allowing prenatal depression screening to better focus preventive intervention to limit PPD emergence. In a previous exploratory study, dexmedetomidine administration in the early postpartum period reduced PPD incidence and was well tolerated.^8^

Dexmedetomidine is a highly selective α_2_-adrenoreceptor (α_2_-AR) agonist commonly used in the perioperative period. Postmortem studies of depressed patients completing suicide show α_2_-AR expression to be increased in several brain regions.^9^ The platelets of patients with PPD also show increased α_2_-AR density, which was reversed by successful antidepressant treatment.^10^ The role of the α_2_-AR is also indicated by preclinical data, with dexmedetomidine significantly improving depression-like behavior in sleep-deprived mice.^11^ Previous data show an α_2_-AR gene alteration to be associated with PPD susceptibility.^12^ Many studies have also confirmed that dexmedetomidine can upregulate brain-derived neurotrophic factor (BDNF),^13,14^ which is closely related to the pathogenesis and prognosis of PPD.^15,16^ Autry et al^17^ also reported that the rapid antidepressive-like effects of ketamine were dependent on the rapid synthesis of BDNF. Overall, alterations in the levels of α_2_-AR and BDNF are closely associated with mood dysregulation, including PPD, with dexmedetomidine showing promise in the regulation of pathophysiological changes in depression as well as possible treatment efficacy in PPD.

To our knowledge, few studies to date have investigated the clinical use of dexmedetomidine in PPD, and none have investigated changes in BDNF. Therefore, we conducted this randomized clinical trial at 2 centers to clarify the role of dexmedetomidine in the prevention of PPD for women with prenatal depression and whether alterations in plasma BDNF levels were evident.

## Methods

### Design and Participants

This randomized, double-blind, placebo-controlled clinical trial was conducted at The Third Xiangya Hospital of Central South University (approximately 2000 cesarean deliveries per year) and Hunan Maternal and Child Health Hospital (approximately 9000 cesarean deliveries per year) from March 28, 2022, to April 16, 2023. The protocol (Supplement 1) was approved by the ethics committees of the 2 study centers. All patients provided written informed consent before participation. We followed the Consolidated Standards of Reporting Trials (CONSORT) reporting guideline for the designing and reporting of this trial.

The participants were parturients who underwent elective cesarean delivery under spinal anesthesia and required postoperative patient-controlled intravenous analgesia (PCIA). Inclusion criteria were as follows: 18 years of age or older, American Society of Anesthesiologists grade II, prenatal depression (defined as a prenatal Edinburgh Postnatal Depression Scale [EPDS] score >9^18,19^), and the ability to reliably communicate with the investigators and follow study instructions. Exclusion criteria included allergy to dexmedetomidine, heart rate of less than 50 beats per minute or presence of cardiac conduction or rhythm abnormalities such as sick sinus syndrome, preoperative hypotension (defined as systolic blood pressure <90 mm Hg), unstable mental illness, and a history of psychotropic substance abuse.

### Randomization and Blinding

Eligible parturients were randomized to receive dexmedetomidine or placebo in a 1:1 ratio by an online central randomization system. Sixty participants in each group were randomly selected for venous blood sampling. Data were collected and managed by independent staff using an electronic data capture system.

Participants in the dexmedetomidine and control groups were kept blinded to their group assignment. This study involved researchers who conducted the evaluation (blinded) and researchers who administered the treatment (unblinded). The unblinded researchers who administered the interventions were not involved in the evaluation of the postoperative outcomes.

### Procedures

A comprehensive preoperative assessment and baseline data collection were conducted. Vital signs were monitored after entering the operating room. After delivery of the infant, participants in the dexmedetomidine group were administered 0.5 μg/kg of dexmedetomidine in 20 mL of 0.9% saline for 10 minutes. The placebo group received 20 mL of 0.9% saline for the same duration. All participants received PCIA immediately after completion of the 10-minute infusion (dexmedetomidine group, dexmedetomidine, 2.0 μg/kg, plus sufentanil, 2.2 μg/kg, diluted in 100 mL; control group, sufentanil, 2.2 μg/kg, diluted in 100 mL). The PCIA was set for continuous infusion at a rate of 2 mL/h for 48 hours. Vital signs, adverse events (AEs), Ramsay Sedation Scale score, and Numerical Rating Scale (NRS) pain score were recorded during the treatment period. Participants’ EPDS scores were followed up by telephone at 7 and 42 days after delivery. If a participant could not be contacted at 7 and/or 42 days after the procedure, the investigator made telephone contact again within 2 days. Failure to make contact was considered a loss to follow-up.

Peripheral venous blood (3 mL) was drawn from participants included in the BDNF analysis before the administration of anesthesia and at the cessation of PCIA. These samples were placed in EDTA-containing anticoagulant tubes and processed within 1 hour by centrifugation (2000*g*, 4 °C, 15 minutes) to separate the plasma. The separated plasma was stored in a freezer at −80 °C for later use. Brain-derived neurotrophic factor and pro-BDNF levels in the plasma were measured by another researcher who was blinded to the group. Measurement of BDNF and pro-BDNF levels in plasma was performed using the sandwich enzyme-linked immunosorbent assay (ELISA) method according to the instructions of the DuoSet ELISA Development Kit (R&D Systems Inc). All samples were analyzed in duplicate. The assay ranges of BDNF and pro-BDNF were 62.5 to 4000 pg/mL and 78.1 to 5000 pg/mL, respectively. The intra-assay and interassay coefficients of variation were less than 10%.

### Measures

The primary outcome was the incidence of positive PPD screening results at 7 and 42 days post partum, defined as a postpartum EPDS score of more than 9.^19^ Secondary outcomes included the incidence of suicidal ideation at 7 and 42 days post partum; EPDS scores at 7 and 42 days post partum; Insomnia Severity Index (ISI) scores at 1, 2, 7, and 42 days post partum; NRS pain score within 48 hours post partum; and plasma BDNF and pro-BDNF levels. Suicidal ideation was determined by item 10 of the EPDS. For suicidal ideation, participants who answered “yes, quite often,” “sometimes,” or “hardly ever” were categorized as “yes.”^20^

Safety assessment included AEs, vital signs, Ramsay Sedation Scale score, and other safety indicators, such as bradycardia, hypotension, dizziness, nausea, vomiting, respiratory depression, and somnolence. All AEs and laboratory values were assessed according to the Common Terminology Criteria for Adverse Events, version 5.0.^21^

### Statistical Analysis

The previous exploratory study^8,22^ found the incidence of positive PPD screening at 42 days post partum in the control group and the dexmedetomidine group to be 29% and 13%, respectively. A 2-sided test with *P* = .05 and a power value of .9 was used to indicate statistical significance. PASS, version 14.0, software (NCSS Statistical Software) was used to estimate the sample size, considering 135 individuals in each group. Considering a dropout rate of approximately 20%, 169 individuals were included in each group.

GraphPad Prism, version 9.3.1 (GraphPad Software Inc), and SPSS Statistics, version 26.0 (IBM Corp), were used for the plots and statistical analyses. We used an intention-to-treat approach that uses participant data regardless of treatment nonadherence or protocol deviation. Continuous variables are expressed as mean (SD) values and median (IQR) values, depending on whether they were normally distributed. Categorical variables are expressed as frequencies and percentages. Measured data were compared between the 2 groups. In the case of normal distribution and homogeneity of variance, the *t* test was used, and in case of skewed distribution, the Mann-Whitney test was used. The χ^2^ test or the Fisher exact test was used to compare the categorical data between the 2 groups. All statistical tests were 2 sided, and *P* ≤ .05 was considered statistically significant.

## Results

**Figure 1.**
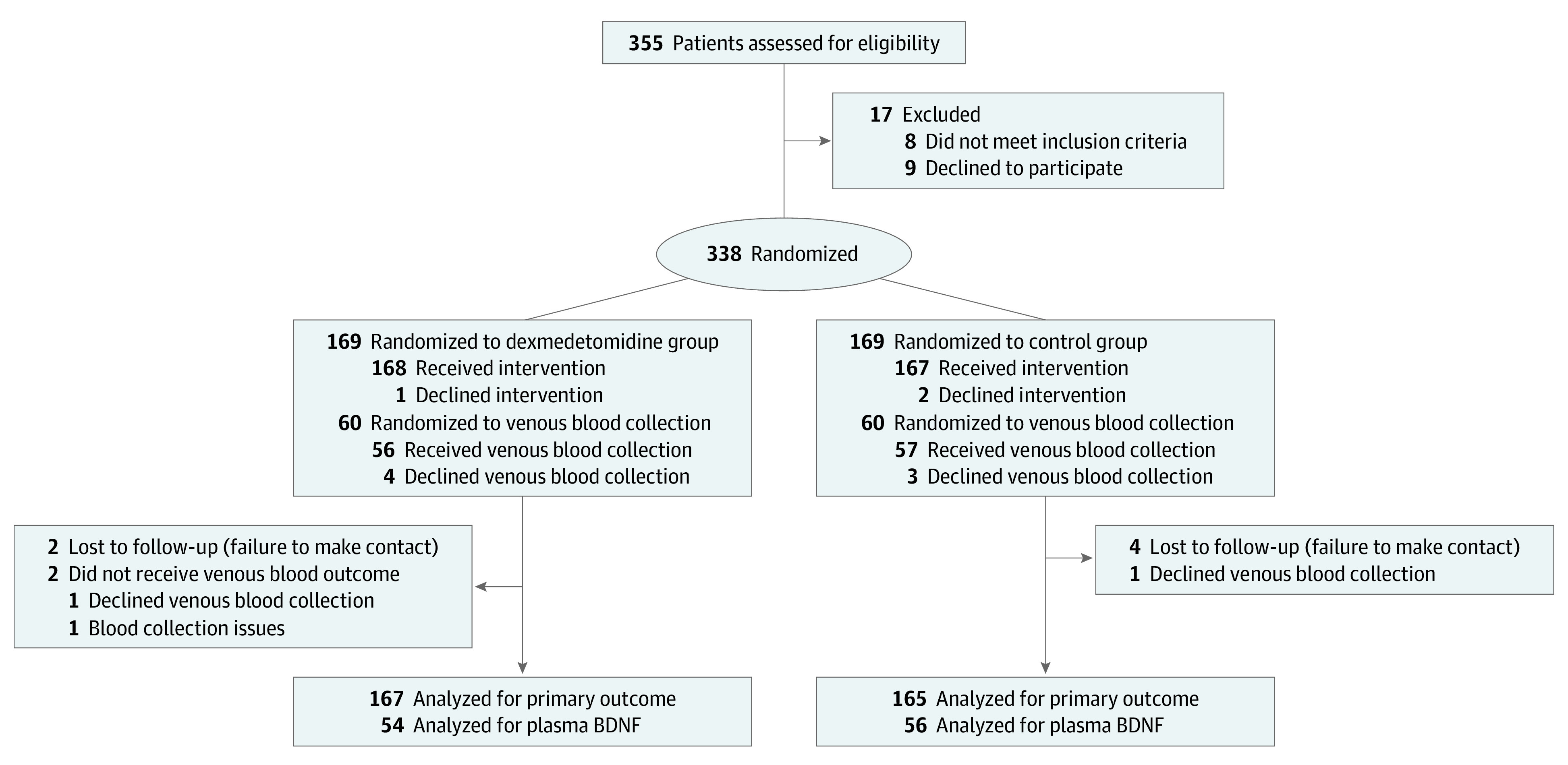
Study Flow Diagram BDNF indicates brain-derived neurotrophic factor.

A total of 355 individuals were assessed for eligibility, and 338 women (mean [SD] age, 31.5 [4.1] years) were randomized to the dexmedetomidine group (n = 169) or the control group (n = 169) (Figure 1). Participants’ baseline characteristics are summarized in Table 1.

**Table 1.** Baseline Characteristics

Characteristic	Control group (n = 169)^a^	Dexmedetomidine group (n = 169)^a^
Age, mean (SD), y	31.5 (4.0)	31.5 (4.2)
BMI, mean (SD)	27.4 (2.8)	27.1 (2.5)
Pressure during pregnancy, No./total No. (%)		
Mild	42/169 (24.9)	44/169 (26.0)
Moderate	75/169 (44.4)	84/169 (49.7)
Severe	52/169 (30.8)	41/169 (24.3)
Mood, No./total No. (%)		
Good	38/169 (22.5)	29/169 (17.2)
Moderate	104/169 (61.5)	108/169 (63.9)
Poor	27/169 (16.0)	32/169 (18.9)
Stressful life events, No./total No. (%)		
Yes	27/169 (16.0)	19/169 (11.2)
No	142/169 (84.0)	150/169 (88.8)
Domestic violence, No./total No. (%)		
No	147/167 (88.0)	146/168 (86.9)
Emotional violence	19/167 (11.4)	22/168 (13.1)
Physical violence	1/167 (0.6)	0
Primipara, No./total No. (%)		
Yes	81/169 (47.9)	67/169 (39.6)
No	88/169 (52.1)	102/169 (60.4)
Full-term pregnancy, No./total No. (%)		
Yes	150/169 (88.8)	156/169 (92.3)
No	19/169 (11.2)	13/169 (7.7)
Planned pregnancy, No./total No. (%)		
Yes	105/169 (62.1)	97/169 (57.4)
No	64/169 (37.9)	72/169 (42.6)
Educational level, No./total No. (%)		
College degree or above	122/167 (73.1)	117/168 (69.6)
High school degree or below	45/167 (26.9)	51/168 (30.4)
Employment, No./total No. (%)		
Yes	103/167 (61.7)	97/168 (57.7)
No	64/167 (38.3)	71/168 (42.3)
Income, No./total No. (%), RMB		
<5000	9/167 (5.4)	5/168 (3.0)
5000-20 000	107/167 (64.1)	126/168 (75.0)
>20 000	51/167 (30.5)	37/168 (22.0)

Abbreviation: BMI, body mass index (calculated as weight in kilograms divided by height in meters squared).

^a^ Percentages have been rounded and may not total 100.

### Primary Outcomes

In the analysis of the primary efficacy outcome in the population with prenatal depression, incidence of positive PPD screening results at 7 and 42 days post partum was significantly decreased in the dexmedetomidine group vs the control group (day 7, 21 of 167 [12.6%] vs 53 of 165 [32.1%]; risk ratio, 0.39 [95% CI, 0.25-0.62]; *P* < .001; day 42, 19 of 167 [11.4%] vs 50 of 165 [30.3%]; risk ratio, 0.38 [95% CI, 0.23-0.61]; *P* < .001) (Table 2).

**Table 2.** Primary and Secondary Outcomes Analysis

Outcome	Control group (n = 169)	Dexmedetomidine group (n = 169)	χ^2^ or *t* Value	*P* value	RR or MD (95% CI)
Incidence of positive PPD screening, No./total No. (%)					
Postpartum day 7	53/165 (32.1)	21/167 (12.6)	χ^2^ = 18.31	<.001	RR, 0.39 (0.25 to 0.62)
Postpartum day 42	50/165 (30.3)	19/167 (11.4)	χ^2^ = 18.06	<.001	RR, 0.38 (0.23 to 0.61)
Suicidal ideation, No./total No. (%)					
Prenatal	34/169 (20.1)	27/169 (16.0)	χ^2^ = 0.98	.32	RR, 0.79 (0.50 to 1.26)
Postpartum day 7	9/165 (5.5)	2/167 (1.2)	χ^2^ = 4.70	.03	RR, 0.22 (0.05 to 1.00)
Postpartum day 42	7/165 (4.2)	4/167 (2.4)	χ^2^ = 0.88	.35	RR, 0.57 (0.17 to 1.89)
Plasma BDNF and pro-BDNF levels, mean (SD), ng/L^a^					
Prenatal BDNF	1491.17 (96.90)	1485.83 (118.46)	*t* = −0.26	.79	MD, −5.34 (−45.65 to 34.97)
Postpartum BDNF	1457.85 (115.59)	1519.38 (132.65)	*t* = 2.60	.01	MD, 61.53 (14.56 to 108.50)
BDNF change value	−35.18 (149.11)	36.39 (159.67)	*t* = 2.43	.02	MD, 71.57 (13.30 to 129.93)
Prenatal pro-BDNF	492.42 (51.24)	498.74 (33.81)	*t* = 0.77	.44	MD, 6.32 (−9.86 to 22.50)
Postpartum pro-BDNF	502.82 (39.75)	489.15 (33.32)	*t* = −1.95	.054	MD, −13.67 (−27.56 to 0.21)
Pro-BDNF change value	9.34 (46.45)	−11.02 (48.69)	*t* = −2.25	.03	MD, −20.36 (−38.35 to –2.38)

Abbreviations: BDNF, brain-derived neurotrophic factor; MD, mean difference; PPD, postpartum depression; RR, risk ratio.

^a^ Prenatal plasma concentration analysis: 57 patients in the control group and 56 patients in the dexmedetomidine group; analysis of postpartum plasma concentration and change value: 56 patients in the control group and 54 patients in the dexmedetomidine group.

### Secondary Outcomes

Compared with the control group, the incidence of suicidal ideation was significantly reduced in the dexmedetomidine group at 7 days post partum. However, there was no significant difference at 42 days post partum (Table 2).

**Figure 2.**
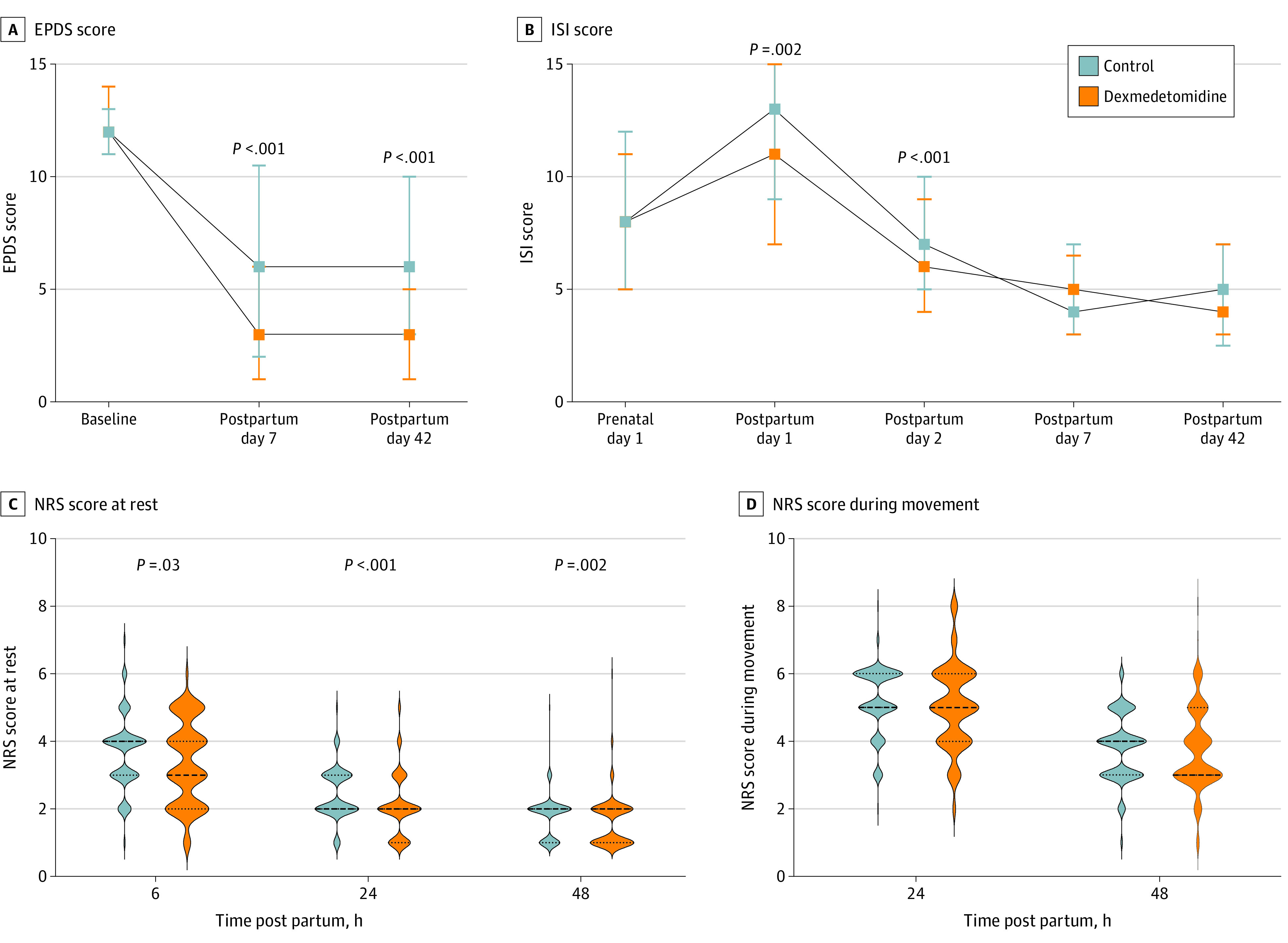
Time Curves for Dexmedetomidine and Control Groups Numerical Rating Scale (NRS) pain scores during movement at 6 hours post partum were missing because patients did not fully recover their motor function. In panels A and B, squares represent medians and error bars represent IQRs. In panels C and D, dashed lines represent medians and dotted lines represent IQRs. EPDS indicates Edinburgh Postnatal Depression Scale; and ISI, Insomnia Severity Index.

The EPDS score and ISI score time curves after preventive dexmedetomidine administration and placebo are shown in Figure 2A and B. The median EPDS score was significantly decreased in the dexmedetomidine group at 7 and 42 days post partum compared with the control group (7 days, 3.0 [IQR, 1.0-6.0] vs 6.0 [IQR, 2.0-10.0]; *P* < .001; 42 days, 3.0 [IQR, 1.0-5.0] vs 6.0 [IQR, 3.0-10.0]; *P* < .001). The median ISI scores of the dexmedetomidine group were significantly decreased at 1 and 2 days post partum compared with the control group (1 day, 11.0 [IQR, 7.0-15.0] vs 13.0 [IQR, 9.0-17.0]; *P* = .002; 2 days, 6.0 [IQR, 4.0-9.0] vs 7.0 [IQR, 5.0-10.0]; *P* < .001). There was no statistically significant difference in ISI scores at 7 and 42 days post partum. The NRS pain intensity time curves for the 2 groups within 48 hours post partum are shown in Figure 2C and D. The median NRS pain scores at rest were significantly decreased in the dexmedetomidine group at 6, 24, and 48 hours post partum compared with the control group (6 hours, 3.0 [IQR, 2.0-4.0] vs 4.0 [IQR, 3.0-4.0]; *P* = .03; 24 hours, 2.0 [IQR, 1.0-2.0] vs 2.0 [IQR, 2.0-3.0]; *P* < .001; 48 hours, 2.0 [IQR, 1.0-2.0] vs 2.0 [IQR, 1.0-2.0]; *P* = .002). There was no significant difference of NRS pain scores during movement.

Plasma BDNF and pro-BDNF levels and their change value for the 2 groups are shown in Table 2. Compared with the control group, the postpartum plasma BDNF level and their change value in the dexmedetomidine group were significantly increased (postpartum plasma BDNF level: mean difference, 61.53 ng/L [95% CI, 14.56-108.50 ng/L]; BDNF change value: mean difference, 71.57 ng/L [95% CI, 13.30-129.93 ng/L]), while the change value of the pro-BDNF was significantly reduced (−20.36 ng/L [95% CI, −38.35 to −2.38 ng/L]). There was no statistically significant difference in pro-BDNF levels after surgery. Further analysis of the correlation between plasma BDNF levels and pro-BDNF levels and positive PPD screening are shown in the eTable in Supplement 2. In the control group of 57 individuals randomized to blood sampling, 17 individuals (29.8%) and 13 individuals (22.8%) developed positive PPD screening results at 7 and 42 days post partum, respectively. The change value of pro-BDNF among participants in the control group with positive PPD screening results was significantly higher than those among participants with nonpositive PPD screening results (mean difference, 33.02 ng/L [95% CI, 4.67-61.37 ng/L] vs 27.65 ng/L [95% CI, 1.40-53.90 ng/L]). In the dexmedetomidine group of 56 individuals randomized to blood sampling, 9 individuals (16.1%) and 5 individuals (8.9%) developed positive PPD screening results at 7 and 42 days post partum, respectively. Brain-derived neurotrophic factor and pro-BDNF were not found to be associated with positive PPD screening results at 7 and 42 days post partum.

### Safety Assessments

There was no significant difference in AEs between the dexmedetomidine and the control groups (46 of 169 [27.2%] vs 33 of 169 [19.5%]; *P* = .10), but the incidence of hypotension increased (31 of 169 [18.3%] vs 16 of 169 [9.5%]; risk ratio, 2.15 [95% CI, 1.13-4.10]; *P* = .02) (Table 3). There was no significant difference in common adverse reactions such as dizziness, bradycardia, nausea, and vomiting. The severity of AEs was 1 to 2 in both groups, except for 1 patient (0.6%) in the dexmedetomidine group, who had a grade 3 AE (classified as a serious AE due to headache from postoperative cerebrospinal fluid loss and prolonged hospital stay). The Ramsay Sedation Scale scores of the 2 groups were close to 2 points (awake, no restlessness) at 6, 24, and 48 hours post partum.

**Table 3.** Analysis of Adverse Event Incidence

Adverse event^a^	Control group, No. (%) (n = 169)	Dexmedetomidine group, No. (%) (n = 169)	χ^2^ Value	*P* value	RR (95% CI)
Adverse events	33 (19.5)	46 (27.2)	2.79	.10	1.39 (0.94-2.07)
Bradycardia	8 (4.7)	17 (10.1)	3.50	.06	2.13 (0.94-4.79)
Bradycardia requiring treatment	0	3 (1.8)	NA	.25^b^	7.00 (0.36-134.49)
Hypotension	16 (9.5)	31 (18.3)	5.56	.02	2.15 (1.13-4.10)
Hypotension requiring treatment	2 (1.2)	8 (4.7)	3.71	.05	4.00 (0.86-18.56)
Nausea	13 (7.7)	14 (8.3)	0.04	.84	1.08 (0.52-2.22)
Vomiting	7 (4.1)	11 (6.5)	0.94	.33	1.57 (0.62-3.96)
Dizziness	11 (6.5)	17 (10.1)	1.40	.24	1.55 (0.75-3.20)
Level 3-5 adverse events	0	1 (0.6)	NA	>.99^b^	3.00 (0.12-73.12)
SAEs	0	1 (0.6)	NA	>.99^b^	3.00 (0.12-73.12)

Abbreviations: NA, not applicable; RR, risk ratio; SAEs, serious adverse events.

^a^ SAEs were identified if daily functions were impaired, or if adverse events were life-threatening and hospitalization or prolonged hospitalization was required. Bradycardia was defined as heart rate less than 60 beats/min. Bradycardia requiring treatment was defined as heart rate less than 50 beats/min. Hypotension was defined as systolic blood pressure less than 90 mm Hg or 20% lower than baseline. Hypotension requiring treatment was defined as systolic blood pressure less than 90 mm Hg or 30% lower than baseline.

^b^ Fisher exact test.

## Discussion

This randomized clinical trial demonstrates the efficacy and safety of dexmedetomidine for women with prenatal depression. Preventive administration of dexmedetomidine in the early postpartum period can reduce positive PPD screening incidence, reduce postoperative pain, and improve sleep quality, with a favorable safety profile. The dexmedetomidine antidepressant effect seems to involve BDNF upregulation.

Dexmedetomidine has central antisympathetic, antianxiety, and sedative effects similar to natural sleep and has certain analgesic effects.^23^ This study indicated that preventive dexmedetomidine administration may decrease positive PPD screening incidence and reduce the postpartum EPDS score among women at high risk for PPD. A study by Metz et al^10^ showed increased α_2_2-AR density in platelets among patients with PPD, which was reversed after successful mood improvement after antidepressant treatment. The involvement of the α_2_-AR in mood dysregulation is also indicated by its upregulation in several brain areas in patients with depression,^9^ as well as by preclinical data showing α_2_-AR gene knockout mice to show depression-like behavior that is not sensitive to tricyclic antidepressants.^24^ This finding is further supported by the data in the present study showing that α_2_-AR activation by dexmedetomidine in the early postpartum period decreased the presence of functional and mood deficits among patients with prenatal depression who were susceptible to PPD. The results of the present study are therefore consistent with those of a previous exploratory study in postpartum women.^8^

The present results indicate that early postpartum dexmedetomidine administration reduces positive PPD screening incidence and significantly increases plasma BDNF levels, while downregulating plasma pro-BDNF levels. Brain-derived neurotrophic factor plays a crucial role in neuronal growth, development, and synaptic plasticity and acts as a neuromodulator.^25^ Pro-BDNF, a precursor of BDNF, has the opposite function and promotes depression and anxiety.^26^ Brain BDNF can cross the blood-brain barrier into the circulation, with brain BDNF level positively correlating with blood BDNF level.^27^ Therefore, the level of BDNF in peripheral blood roughly mirrors its level in the central nervous system. Both clinical and preclinical investigations have shown dexmedetomidine to upregulate BDNF expression while downregulating pro-BDNF.^13,14,28^ A clinical study by Lee et al^16^ showed that the plasma BDNF level of women with postpartum depression decreased significantly, which was reversed on mood recovery. A study by Gao et al^15^ of 340 Chinese women also indicated that the decrease in prenatal serum BDNF level was closely related to PPD occurrence within 3 months and could be used as a biomarker to predict PPD. Other studies of patients with depression show similar results to those in the present study.^29^ All of these results are consistent with our study.

Further analysis showed that in the control group, the change value of pro-BDNF was significantly higher among patients with a positive PPD screening vs those with a nonpositive PPD screening at 7 and 42 days post partum, with BDNF showing a decreasing trend. However, no significant correlation was evident between positive PPD screening occurrence and BDNF or pro-BDNF levels in the dexmedetomidine group. This finding may be related to the insufficient sample size due to the lower number of positive PPD screenings in the dexmedetomidine group. Although a complexity of processes may be linked to the alterations in BDNF and pro-BDNF in this study, the changes in plasma BDNF and especially pro-BDNF may serve as a readily accessible, sensitive predictor of PPD.

Suicide is the leading cause of perinatal death,^30^ highlighting the importance of suicidal ideation screening of mothers at high risk to initiate clinical intervention.^31^ Our study showed that dexmedetomidine significantly reduced suicidal ideation at 7 days post partum but not 42 days post partum, unlike in the previous exploratory study in which suicide ideation was decreased by dexmedetomidine at both time points.^8^ This discrepancy could be attributed to a lower incidence of suicidal ideation post partum in the study sample or an insufficient sample size. The emphasis of the present study on women at high risk of PPD may also have influenced these results due to the 2-day postoperative drug treatment being insufficient at the later time point for more severe PPD presentations. Further studies will be required to clarify.

Sleep disorders have been identified as potential contributors to PPD and may increase suicidal ideation among mothers.^32,33,34^ Our studies found significant improvement in sleep quality among parturients during dexmedetomidine infusion, mirroring the findings of Oxlund et al^35^ and Huang et al.^36^ The sedative effect of dexmedetomidine, which is similar to natural sleep and acts on the locus coeruleus α_2_-AR, may contribute to this process.^37^ Further evidence for this hypothesis is provided by the results of Moon et al,^11^ who demonstrated that dexmedetomidine improved depression-like behavior in sleep-deprived mice. However, our results show that dexmedetomidine improved sleep only within 48 hours during infusion, and whether dexmedetomidine-improved sleep is related to PPD needs further clarification.

Uncontrolled postoperative pain not only seriously affects the quality of life and function of parturients, it also increases PPD incidence.^38,39^ Our study showed that postoperative dexmedetomidine administration improved maternal postoperative pain at rest, with limited control of pain during movement. Dexmedetomidine is commonly used as an adjuvant analgesic in clinical practice and has a good synergistic effect with opioids without causing respiratory depression.^40,41^ The unremarkable improvement in pain on movement can be attributed to the dose we administered (0.04 μg/kg/h), which is much lower compared with the standard clinical dose (0.6 μg/kg/h).^42,43^

In this study, a loading dose of dexmedetomidine, 0.5 μg/kg combined with a maintenance low-dose PCIA was used to make up for the short duration of drug effect after a single administration, to reduce the adverse effects related to blood concentration dependence,^40^ and to facilitate postoperative management. The most common adverse effects of dexmedetomidine in clinical practice are decreased heart rate, decreased blood pressure, and dizziness.^40^ The present study found hypotension incidence to be significantly increased among the dexmedetomidine group vs the control group, although interventions were rarely required. No statistical difference was found in the incidence of bradycardia between the 2 groups, which was inconsistent with the study results of Bao et al.^44^ It is possible that the current sample size was insufficient to detect a difference statistically. The incidence of AEs did not differ significantly between the 2 groups.

### Limitations

Our study has several limitations. First, we randomly selected only a small number of patients to monitor BDNF levels, and the results from 7 days and 42 days post partum were not available. Further studies are needed to verify the role of BDNF in the antidepressant effect of dexmedetomidine. Second, the dose of dexmedetomidine was determined based on previous single-dose studies. The optimal effective dose of dexmedetomidine needs further research. Third, the EPDS score cannot diagnose PPD; it can only be used as a positive screening tool for PPD.

## Conclusions

In this randomized clinical trial, preventive administration of dexmedetomidine in the early postpartum period reduced the incidence of positive PPD screening, reduced postoperative pain, improved sleep quality, and maintained a favorable safety profile. Its antidepressant effect may be related to BDNF upregulation and/or downregulation of pro-BDNF.

## Notes

Supplement 1.
Trial Protocol

Supplement 2. **eTable.** Correlation Analysis Between Plasma BDNF and Pro-BDNF Levels and Positive PPD Screen

Supplement 3.
Data Sharing Statement
