# Supplementary material for: Effect of Dexmedetomidine on Postpartum Depression in Women With Prenatal Depression: A Randomized Clinical Trial
Source: JAMA Netw Open. 2024 Jan 25;7(1):e2353252. doi: 10.1001/jamanetworkopen.2023.53252 (PMC10811555; doi:10.1001/jamanetworkopen.2023.53252)
Supplement: Supplement 1. — Trial Protocol [file jamanetwopen-e2353252-s001.pdf]

# Effect of perioperative dexmedetomidine intervention on postpartum depression in women undergoing cesarean section: a randomized, double-blind, controlled clinical trial

Sponsoring Institutions: The Third Xiangya Hospital of Central South University

Group Institutions: The Third Xiangya Hospital of Central South University

Research Institutions: The Third Xiangya Hospital, Central South University

Principal study leaders: Saiying Wang

Version number: 2.1

Principal study leaders:

Date of signature:

CONTENTS

Abstract of the study protocol.............................................................. 3

Text of the study........................................................................................ 4

1 Research Background.............................................................................. 4

2 Objectives………….............................................................................. 5

3 Trial design.............................................................................................. 5

4 Content of the study................................................................................ 7

5 Efficacy measurements…………........................................................... 8

6 Safety assessment......................................................................................9

7 Statistical analysis.................................................................................... 9

8 Data integrity and quality assurance…….............................................. 10

9 Statement of Ethics...................................................................................11

## Abstract of the study protocol

| |
|---|
| **Title**: Effect of perioperative dexmedetomidine intervention on postpartum depression in women undergoing cesarean section: a randomized, double-blind, controlled clinical trial |
| **Objectives**: To investigate the effects of Preventive administration of dexmedetomidine (DEX) in the early postpartum period on postpartum depressive symptoms (PDS) in parturients with prenatal depression. In addition, the possible mechanisms of the antidepressant effect of DEX will be investigated. |
| **Trial Design:** Prospective randomized double-blind placebo-controlled trial.<br><br>**Study procedure:** Participants were randomly assigned to the DEX or control group. In the DEX and control groups, 5ug/kg and normal saline were infused after delivery. After infusion, DEX plus sufentanil or sufentanil was administered via patient-controlled intravenous analgesia (PCIA). Vital signs (including heart rate, blood pressure, respiratory rate, blood oxygen saturation), AEs, sedation score (Ramsay Sedation Score) and NRS pain score were recorded during the treatment period. Subjects' EPDS scores were followed up by telephone 7 and 42 days after delivery. BDNF and pro-BDNF levels in the plasma were measured by another researcher who was blinded.<br><br>**Inclusion criteria**: Women who underwent elective caesarean section under spinal anesthesia and required postoperative PCIA; Over 18 years of age; American Society of Anesthesiologists (ASA) grade II; Prenatal Edinburgh Postnatal Depression Scale (EPDS) score > 9; and the ability to reliably communicate with the investigators and follow study instructions.<br><br>**Exclusion criteria**: Allergy to dexmedetomidine; Heart rate of less than 50 beats per minute (bpm) or presence of cardiac conduction or rhythm abnormalities such as sick sinus syndrome; Preoperative hypotension (defined as systolic blood pressure of less than 90 mmHg); Unstable mental illness A history of psychotropic substance abuse. |
| **Statistical analysis**<br>SPSS Statistics 26.0 (IBM Corp., Armonk, NY, USA) and Graphpad Prism 9.3.1 (Graphpad Software, Inc., California) were used for the statistical analyses and plots. Continuous variables were described as means (standard deviation, SD) and medians (interquartile range, IQR), depending on whether they were normally distributed or not. Categorical variables were described as frequencies (percentages). Logistic regression models were used to compare the incidence of PDS at 7 and 42 days postpartum between the two groups. Models were adjusted for potential confounders. Measured data were compared between the two groups. In case of normal distribution and homogeneity of variance, the t-test was used, and in case of skewed distribution, the Mann-Whitney U-test was used. The chi-square test or Fisher's exact test was used to compare the categorical data between the two groups. All statistical tests were two-sided and p-value ≤0.05 was considered statistically significant. |

Text of the study

# 1. **Background**

## 1.1 Postpartum depression is of great harm, and its prevention and treatment is of great significance.

Postpartum depressive symptoms (PDS) are the most common mental and psychological disorder in postpartum women, with an incidence of about 10-20%. The first six weeks after delivery may be a high-risk period for depression. The main symptoms include depressed mood, sadness, insomnia, anxiety, irritability and slow thinking. Some even have the idea of suicide and harming the baby PDS has significant and far-reaching effects that not only harm the physical and mental health of mothers, but also seriously affect the family, marriage and children's intellectual, social and emotional development and behavior. Although the incidence of postpartum depression is high, the awareness rate of postpartum depression is low, the rate of seeking health care is low, only a few people have access to psychological treatment, and most people are unwilling to take antidepressant drugs during pregnancy or breastfeeding. Therefore, it is of great significance to study the occurrence, prevention and treatment of PDS.

## 1.2 α2-adrenergic receptor (a2-AR) is closely related to postpartum depression

Clinical evidence suggests a relationship between the central noradrenergic system and fear/anxiety states or depression. The "catecholamine hypothesis" of mood disorders emphasizes that depression is a consequence of a relative deficiency of brain catecholamines, particularly norepinephrine (NE). a2A-AR activation is thought to play a protective role in depression as well as other psychiatric disorders including opioid abuse and dependence. Studies in suicidal patients with depression have shown that α2AR expression is increased in several parts of the brain [19]. The clinical study by Metz et al [20] also showed that the α2AR density and affinity of platelets from PDS patients was increased and the α2AR density and affinity of platelets reversed while symptoms were relieved after antidepressant treatment.

## 1.3 Antidepressant effect of dexmedetomidine

Dexmedetomidine (DEX) is a highly selective α2-AR agonist commonly used in clinical practice. It has central antisympathetic, antianxiety, and sedative effects similar

to natural sleep, and has certain analgesic effects. This effect may help maternal avoid perioperative stress and improve perioperative comfort without the risk of respiratory depression. Many studies have confirmed that DEX can upregulate BDNF, which is closely related to the occurrence and prognosis of PDS. DEX can also be used as an adjuvant analgesia in the perioperative period of cesarean section to reduce the incidence of postpartum depression.

1.4 Prenatal depression is a risk factor for PDS

Antenatal depression should be paid special attention to and prevention should be strengthened. Numerous studies have shown that postpartum depression is often associated with perinatal stress, low economic income, genetic factors, and other psychological and social predisposing factors. Prenatal depression is one of the most important risk factors for postpartum depression and has a certain predictive effect on postpartum depression. Therefore, this study selected this population as the observation object, hoping to obtain objective and reliable evidence through this study, and to provide guidance for the clinical application of dexmedetomidine in patients with antenatal depression and the prevention and treatment of postpartum depression.

## 2. **Objectives**

To investigate the effects of Preventive administration of dexmedetomidine (DEX) in the early postpartum period on postpartum depressive symptoms (PDS) in parturients with prenatal depression. In addition, the possible mechanisms of the antidepressant effect of DEX will be investigated.

## 3. **Trial Design**

### 3.1. Sample size calculation

This was a randomized controlled clinical trial with dexmedetomidine as the intervention group and placebo as the control group, and the outcome measure was the incidence of postpartum depression 42 days postpartum. According to the previous exploratory study, the incidence of PDS in the control group and the DEX group was 29% and 13%, respectively. A two-sided test with a p-value of 0.05 and a power value of 0.9 was used, with the control group and the DEX group in a 1:1 ratio. PASS 14.0 software was used to estimate the sample size, considering 135 cases in each group. Considering a dropout rate of about 20%, a total of 338 subjects were to be enrolled in this study, 169 in the control group and 169 in the DEX group.

### 3.2 Randomization

Central computer-generated group randomization was used, in which all eligible participants were assigned a unique randomization number that was allocated sequentially according to a predetermined randomization scheme.

3.3 Blinding

The trial was conducted in a double-blind manner, and placebo was saline that was identical in appearance, size, and color to dexmedetomidine but did not have any pharmacological effects. The non-blind anesthesiologists ensured the grouping of the subjects. The perioperative observation personnel and the postoperative follow-up personnel were unaware of the treatment received. The maintenance of blinding throughout the trial was essential. In the event of a medical emergency during the administration of the study drug, knowledge of the treatment randomization was necessary for the appropriate treatment of the subject, and emergency unblinding was performed.

3.4. Placebo control

In this study, normal saline was used as placebo without any pharmacological effects.

3.5 CONSORT flow diagram

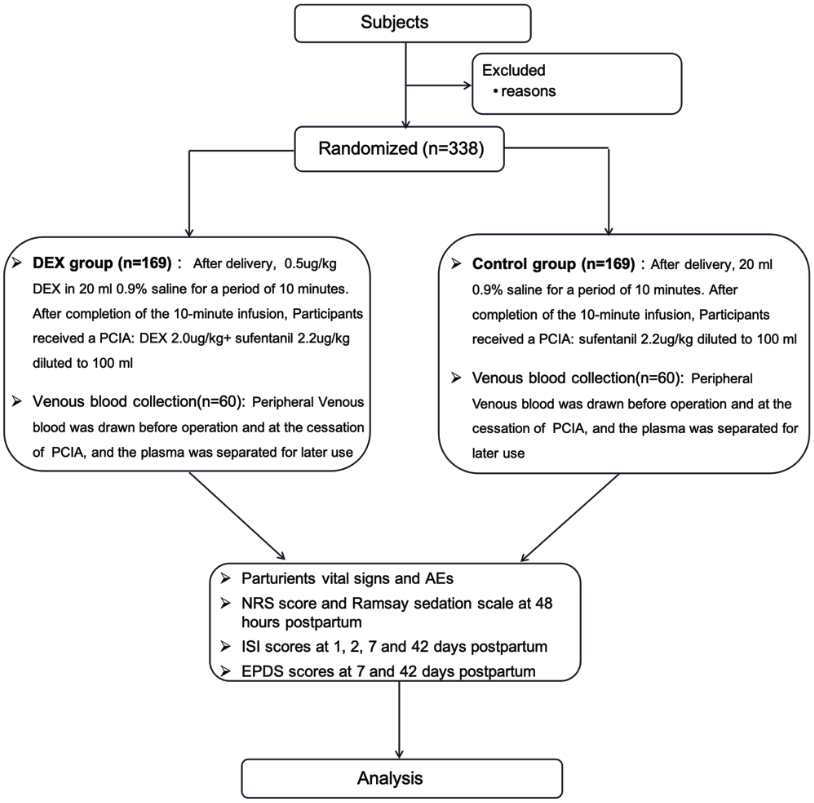

## 4. **Content of the study**

### 4.1. Study Procedures

Before and during surgery : A comprehensive preoperative assessment and baseline data collection was conducted. Baseline information included age, height, weight, stress during pregnancy (mild, moderate, severe), mood during pregnancy (good, moderate, poor), stressful life events, domestic violence during pregnancy, primipara, planned or unwanted pregnancy, maternal education level, employment or not, and monthly household income (Chinese renminbi, RMB). The EPDS was used to assess prenatal depression during pregnancy, defined by the EPDS score > 9. After entering the operating room, intravenous access was established, vital signs were monitored, and oxygen was administered through a nasal cannula at a rate of 2 l/min. Spinal anesthesia was administered by an experienced anesthesiologist. After successful puncture at L2-3 or L3-4, ropivacaine (15 mg), fentanyl (25 ug) and 10% glucose (0.5 ml) were administered into the subarachnoid space. After successful anesthesia, the operating table was transitioned to a 20-30 degree left lateral position to reduce the risk of hypotensive syndrome in the supine position. After delivery of the infant, participants in the DEX group were administered 0.5ug/kg DEX in 20 ml 0.9% saline for a period of 10 minutes. The placebo group received 20 ml of 0.9% saline for the same duration. Vital signs (including heart rate, blood pressure, respiratory rate, blood oxygen saturation), AEs, sedation score (Ramsay Sedation Score) and NRS pain score were recorded during the treatment period.

Postoperative analgesia: All participants received a PCIA immediately after completion of the 10-minute infusion. DEX group: dexmedetomidine 2.0ug/kg+ sufentanil 2.2ug/kg diluted to 100 ml; Control group: sufentanil 2.2ug/kg diluted to 100 ml. The PCIA was set for continuous infusion at a rate of 2 ml/h for 48 hours. Following surgery, the presence of participant pain and the NRS pain score at rest ≥4, the appropriate amount of analgesic could be routinely given and recorded as rescue analgesia.

Analysis of plasma BDNF and pro-BDNF: Peripheral venous blood (3ml) was drawn from participants included in the BDNF analysis before the administration of anesthesia and at the cessation of PCIA. These samples were placed in EDTA-containing anticoagulant tubes and processed within one hour by centrifugation at low temperature (2000g, 4°C, 15min) to separate the plasma. The separated plasma was stored in a

freezer at − 80°C for later use. BDNF and pro-BDNF levels in the plasma were measured by another researcher who was blinded to the group source of the sample. Measurement of BDNF and pro-BDNF levels in plasma was performed according to the instructions of the Kit. All samples were analyzed in duplicate.

Inclusion criteria: Women who underwent elective caesarean section under spinal anesthesia and required postoperative PCIA; Over 18 years of age; American Society of Anesthesiologists (ASA) grade II; Prenatal Edinburgh Postnatal Depression Scale (EPDS) score > 9; and the ability to reliably communicate with the investigators and follow study instructions.

Exclusion criteria: Allergy to dexmedetomidine; Heart rate of less than 50 beats per minute (bpm) or presence of cardiac conduction or rhythm abnormalities such as sick sinus syndrome; Preoperative hypotension (defined as systolic blood pressure of less than 90 mmHg); Unstable mental illness A history of psychotropic substance abuse.

4.3. Study period: March 2022 to May 2023

4.4. Follow-up

After the operation, the women were sent back to the ward and the analgesia pump was connected to work. The analgesia pump was used until 48 hours after the operation. The vital signs, adverse reactions, Ramsay sedation score, NRS pain score and Insomnia Severity Index (ISI) were followed up by trained observers at 6-8h, 24 h and 48 h after the operation. Participants' EPDS and ISI scores were followed up by telephone at 7 and 42 days after delivery. If a participant could not be contacted at 7 and/or 42 days after the procedure, the investigator made telephone contact again within 2 days. Failure to make contact was considered a loss to follow-up.

5. **Efficacy measurements**

The Chinese version of Edinburgh Postnatal Depression Scale (EPDS) was used to assess PDS at 42 days postpartum. PDS was defined as postpartum EPDS score > 9. Suicidal ideation was determined by item 10 of the EPDS. For suicidal ideation, participants who answered “yes, quite often",” “sometimes” or “hardly never" were categorized as “yes",” while they were categorized as “no” if they answered “never”. Postpartum pain was evaluated by numerical rating scale (NRS). Insomnia severity index (ISI) was used to evaluate postpartum sleep quality. Ramsay sedation scale was used for postpartum

sedation.

5.1 The primary outcome

The incidence of PDS at 7 and 42 days postpartum.

5.2 Secondary efficacy endpoints

The incidence of suicidal ideation at 7 and 42 days postpartum; EPDS scores at 7 and 42 days postpartum; NRS pain score within 48 hours postpartum; ISI scores at 1, 2, 7 and 42 days postpartum; Plasma BDNF and pro-BDNF levels.

6. **Safety assessment**

Dexmedetomidine is a marketed drug with good safety and controllable adverse reactions. Safety assessment included AEs, vital signs, Ramsay sedation score and other safety indicators. All AEs and laboratory values were assessed according to the Common Terminology Criteria for Adverse Events version 5.0. Serious adverse events (SAEs) were identified if daily functions were impaired, or life-threatening and hospitalization or prolonged hospitalization was required. Respiratory depression was defined as pulse oxygen saturation ($SpO_2$) < 90%. Hypotension was defined as systolic blood pressure (SBP) < 90 mmHg or 20% lower than baseline. Hypotension requiring treatment was defined as systolic blood pressure (SBP) < 90mmHg or 30% lower than baseline. Bradycardia was defined as heart rate < 60 bpm. Bradycardia requiring treatment was defined as heart rate < 50 bpm.

7. **Statistical analysis**

SPSS Statistics 26.0 (IBM Corp., Armonk, NY, USA) and Graphpad Prism 9.3.1 (Graphpad Software, Inc., California) were used for the statistical analyses and plots. Continuous variables were described as means (standard deviation, SD) and medians (interquartile range, IQR), depending on whether they were normally distributed or not. Categorical variables were described as frequencies (percentages). Logistic regression models were used to compare the incidence of PDS at 7 and 42 days postpartum between the two groups. Models were adjusted for potential confounders. Measured data were compared between the two groups. In case of normal distribution and homogeneity of variance, the t-test was used, and in case of skewed distribution, the Mann-Whitney U-test was used. The chi-square test or Fisher's exact test was used to compare the categorical data between the two groups. All statistical tests were two-

sided and p-value ≤0.05 was considered statistically significant.

## 8. **Data integrity and quality assurance**

### 8.1 Data recording and management

Investigators must vouch for the authenticity, completeness, and accuracy of the data. All items in the original records of the study should be clearly filled in with a black marker, no empty items or missing items (slash lines in the blanks without records). Any corrections should only be made with a horizontal line, the corrected data should be circumscribed, signed and dated by the investigator, and the original clinical original records should not be blotted out or overwritten. Any corrections should only be made with a horizontal line, the corrected data should be circumscribed, signed and dated by the investigator, and the original clinical original records should not be blotted out or overwritten. The investigator must retain the original clinical records and informed consent of each participant enrolled in the trial. The original clinical records should contain all demographic and treatment information, including laboratory data, electrocardiograms, etc., as well as the subject's concomitant medications and adverse events. All information on the case report form must be derived from original clinical records. Data regarding subjects on the case report form should be recorded in code form, with subjects identified only by trial number or their initials. Investigators should complete clinical original records and case report forms in a timely manner so that the monitor can check the condition of the subjects throughout the study.

Clinical original records should document all information required by the study protocol, and any omissions should be justified. Monitors check records for completeness, accuracy, and consistency, as well as investigator signatures. To ensure the quality of clinical data for all subjects and for each study unit, data from the CRF will be checked electronically and manually.

### 8.2 Record keeping

All clinical research documents, including clinical research protocol and its amendments, ethical approval, clinical original records and laboratory examination records, case report forms, clinical study reports, etc., should be uniformly preserved in the research institution. All clinical research documents should be maintained in secure records for the period defined by institutional or site policy.

### 8.3 Quality Assurance

Investigators should perform their own duties, strictly follow the clinical trial protocol, and adopt standard operating procedures to ensure the implementation of the quality control and quality assurance system of clinical trials. All relevant observations and findings in clinical trials should be verified, and quality control must be carried out at every stage of data processing to ensure that the data are complete, accurate, true and reliable.

9. **Ethical Statement**

The clinical study will follow the World Medical Assembly Declaration of Helsinki and other relevant regulations. The clinical study was carried out after approval of the trial protocol by the ethics committee prior to the initiation of the study. Before each subject is enrolled in this study, the investigator is responsible to provide the subject or his/her representative with a complete and comprehensive introduction of the purpose, procedures and possible risks of this study, and sign a written informed consent form. The investigator should let the subject know that they have the right to withdraw from this study at any time. The personal privacy and data confidentiality of the subjects will be protected during the study.
