# Supplementary material for: Effect of Dexmedetomidine on Postpartum Depression in Women With Prenatal Depression: A Randomized Clinical Trial
Source: JAMA Netw Open. 2024 Jan 25;7(1):e2353252. doi: 10.1001/jamanetworkopen.2023.53252 (PMC10811555; doi:10.1001/jamanetworkopen.2023.53252)
Supplement: Supplement 2. — eTable. Correlation Analysis Between Plasma BDNF and Pro-BDNF Levels and Positive PPD Screen [file jamanetwopen-e2353252-s002.pdf]

Supplementary Online Content

Zhou Y, Bai Z, Zhang W, et al. Effect of dexmedetomidine on postpartum depression in women with prenatal depression: a randomized clinical trial. *JAMA Netw Open.* 2024;7(1):e2353252. doi:10.1001/jamanetworkopen.2023.53252

eTable. Correlation Analysis Between Plasma BDNF and Pro-BDNF Levels and Positive PPD Screen

This supplementary material has been provided by the authors to give readers additional information about their work.

© 2024 Zhou Y et al. *JAMA Network Open.*

eTable. Correlation Analysis Between Plasma BDNF and Pro-BDNF Levels and Positive PPD Screen

Plasma levels	non-positive PPD screen		positive PPD screen		t	*p* - value	MD (95% CI)
	n	mean (SD)	n	mean (SD)			
PPD at postpartum 7 days in the control group							
Prenatal BDNF	39	1479.67(99.49)	18	1516.06(88.57)	1.33	0.19	36.39(-18.57–91.35)
Postpartum BDNF	39	1466.88(107.82)	17	1437.15(132.89)	-0.88	0.38	-29.73(-97.21–37.75)
BDNF change value	39	-12.80(144.03)	17	-86.52(152.10)	-1.73	0.09	-73.72(-159.07–11.62)
Prenatal Pro-BDNF	39	500.96(48.97)	18	473.92(52.51)	-1.90	0.06	-27.05(-55.65–1.56)
Postpartum Pro-BDNF	39	501.91(39.74)	17	504.91(40.92)	0.26	0.79	3.00(-20.36–26.36)
Pro-BDNF change value	39	0.95(45.30)	17	28.60(44.45)	2.11	0.04	27.65(1.40–53.90)
Positive PPD screen at postpartum 42 days in the control group							
Prenatal BDNF	44	1487.67(96.07)	13	1503.00(102.73)	0.50	0.62	15.32(-46.39–77.05)
Postpartum BDNF	43	1466.39(114.00)	13	1429.61(120.91)	-1.01	0.32	-36.78(-110.12–36.55)
BDNF change value	43	-23.62(139.47)	13	-73.39(178.20)	-1.06	0.30	-49.77(-144.29–44.75)
Prenatal Pro-BDNF	44	499.05(51.34)	13	469.98(45.82)	-1.84	0.07	-29.07(-60.82–2.68)
Postpartum Pro-BDNF	43	502.26(42.02)	13	504.68(32.52)	0.19	0.85	2.42(-23.03–27.87)
Pro-BDNF change value	43	1.68(43.12)	13	34.70(49.74)	2.34	0.02	33.02(4.67–61.37)
Positive PPD screen at postpartum 7 days in the dexmedetomidine group							
Prenatal BDNF	47	1479.21(114.22)	9	1520.37(140.94)	0.95	0.34	41.15(-45.33–127.64)
Postpartum BDNF	45	1521.82(131.26)	9	1507.22(146.99)	-0.30	0.77	-14.59(-112.63–83.44)
BDNF change value	45	46.30(165.68)	9	-13.14(120.88)	-1.02	0.31	-59.45(-176.40–57.51)
Prenatal Pro-BDNF	47	498.27(33.54)	9	501.19(37.18)	0.24	0.82	2.92(-21.96–27.80)
Postpartum Pro-BDNF	45	490.66(35.68)	9	481.57(16.62)	-1.18	0.24	-9.10(-24.89–6.70)
Pro-BDNF change value	45	-9.30(51.32)	9	-19.63(33.46)	-0.58	0.57	-10.32(-46.23–25.58)
Positive PPD screen at postpartum 42 days in the dexmedetomidine group							
Prenatal BDNF	51	1485.53(119.16)	5	1488.88(124.36)	0.06	0.95	3.36(-108.97–115.68)
Postpartum BDNF	49	1513.40(127.27)	5	1578.00(184.49)	1.04	0.30	64.60(-60.27–189.47)
BDNF change value	49	31.01(150.67)	5	89.12(248.13)	0.51	0.63	58.11(-247.12–363.34)
Prenatal Pro-BDNF	51	498.83(35.00)	5	497.90(20.12)	-0.06	0.95	-0.93(-32.98–31.13)
Postpartum Pro-BDNF	49	491.32(33.93)	5	467.83(16.59)	-1.52	0.16	-23.49(-54.51–7.52)
Pro-BDNF change value	49	-9.08(50.15)	5	-30.07(26.98)	-0.92	0.36	-20.99(-66.93–24.95)

© 2024 Zhou Y et al. *JAMA Network Open.*
