# Supplementary material for: Effect of Dexmedetomidine on Postpartum Depression in Women With Prenatal Depression: A Randomized Clinical Trial
Source: JAMA Netw Open. 2024 Jan 25;7(1):e2353252. doi: 10.1001/jamanetworkopen.2023.53252 (PMC10811555; doi:10.1001/jamanetworkopen.2023.53252)
Supplement: Supplement 3. — Data Sharing Statement [file jamanetwopen-e2353252-s003.pdf]

Data Sharing Statement

Zhou. Effect of Dexmedetomidine on Postpartum Depression in Women With Prenatal Depression. *JAMA Netw Open*. Published January 25, 2024.
doi:10.1001/jamanetworkopen.2023.53252

Data
Data available: Yes
Data types: Deidentified participant data
How to access data: duankaiming@126.com ; zwyhyll@163.com
When available: With publication

Supporting Documents
Document types: Statistical/analytic code
How to access documents: duankaiming@126.com ; zwyhyll@163.com
When available: With publication

Additional Information
Who can access the data: researchers whose proposed use of the data has been approved
Types of analyses: Non-commercial purposes
Mechanisms of data availability: With investigator support and with a signed data access agreement
